# Expression of a Novel Antimicrobial Peptide Penaeidin4-1 in Creeping Bentgrass (*Agrostis stolonifera* L.) Enhances Plant Fungal Disease Resistance

**DOI:** 10.1371/journal.pone.0024677

**Published:** 2011-09-12

**Authors:** Man Zhou, Qian Hu, Zhigang Li, Dayong Li, Chin-Fu Chen, Hong Luo

**Affiliations:** Department of Genetics and Biochemistry, Clemson University, Clemson, South Carolina, United States of America; Cairo University, Egypt

## Abstract

**Background:**

Turfgrass species are agriculturally and economically important perennial crops. Turfgrass species are highly susceptible to a wide range of fungal pathogens. Dollar spot and brown patch, two important diseases caused by fungal pathogens *Sclerotinia homoecarpa* and *Rhizoctonia solani*, respectively, are among the most severe turfgrass diseases. Currently, turf fungal disease control mainly relies on fungicide treatments, which raises many concerns for human health and the environment. Antimicrobial peptides found in various organisms play an important role in innate immune response.

**Methodology/Principal Findings:**

The antimicrobial peptide - Penaeidin4-1 (Pen4-1) from the shrimp, *Litopenaeus setiferus* has been reported to possess in vitro antifungal and antibacterial activities against various economically important fungal and bacterial pathogens. In this study, we have studied the feasibility of using this novel peptide for engineering enhanced disease resistance into creeping bentgrass plants (*Agrostis stolonifera* L., cv. Penn A-4). Two DNA constructs were prepared containing either the coding sequence of a single peptide, Pen4-1 or the DNA sequence coding for the transit signal peptide of the secreted tobacco AP24 protein translationally fused to the Pen4-1 coding sequence. A maize ubiquitin promoter was used in both constructs to drive gene expression. Transgenic turfgrass plants containing different DNA constructs were generated by *Agrobacterium*-mediated transformation and analyzed for transgene insertion and expression. In replicated in vitro and in vivo experiments under controlled environments, transgenic plants exhibited significantly enhanced resistance to dollar spot and brown patch, the two major fungal diseases in turfgrass. The targeting of Pen4-1 to endoplasmic reticulum by the transit peptide of AP24 protein did not significantly impact disease resistance in transgenic plants.

**Conclusion/Significance:**

Our results demonstrate the effectiveness of Pen4-1 in a perennial species against fungal pathogens and suggest a potential strategy for engineering broad-spectrum fungal disease resistance in crop species.

## Introduction

Turfgrasses, agriculturally and economically important crop species, are used worldwide for lawns of buildings, roadsides, athletic and recreational fields providing numerous benefits including reducing soil erosion, trapping dust and pollutants, moderating temperature, safer playing grounds and beautifying the environment [1], [2]. There are more than 50 million acres of turfgrass and 16,000 golf courses in the US alone, and the turfgrass industry is a multibillion dollar business annually [1], [2]. Turfgrass species are highly susceptible to a wide range of fungal pathogens. Dollar spot and brown patch, two important diseases caused by fungal pathogens *Sclerotinia homoecarpa* and *Rhizoctonia solani* respectively, are among the most severe and frequently occurring diseases on turfgrass lawns in the summer [3], [4]. Currently, fungicides are commonly applied to control fungal diseases. This raises concerns about the potential emergence of new pathogen strains as a result of intensive use of chemicals [5]–[7]. Resistance to some major classes of fungicides such as benzimidazoles, demethylation inhibitors (DMIs), Qo respiration inhibitors (QoIs) and dicarboximides (DCFs) has been detected in many phytopathogenic fungi species [8]. For example, large scale agricultural use of DMIs since 1970s has led to the emergence of resistant genotypes of several phytopathogenic fungi impacting different crop and fruit species including turfgrass [6], [8]–[10]. Similarly, benzimidazole-resistant genotypes were also identified in *Monilinia fruccticola*, *Penicillium expansum*, *Botrytis cinerea*, *Helminthosporium solani* and *sclerotinia homoeocarpa.* [6], [8], [11]–[14]. Therefore, the problem of emergent new resistant pathogen strains and the negative long-term impacts of fungicides on human health and the environment have both driven the search for new alternatives for the currently used chemicals [5], [15]. It is desirable that new cultivars be developed that present sustainable resistance to a broad range of pathogens and are safe for the environment or human consumption [5], [16].

Antimicrobial peptides (AMPs) found in various organisms play an important role in innate immune response [16]–[20], providing good candidates for use in plants for enhanced disease resistance. AMPs are short sequence peptides with generally fewer than 50 amino acid residues, most of which have antimicrobial activity against a broad spectrum of pathogens. They are a first line of defense in plants and animals and resistance against them is much less observed compared with current antibiotics [16]. AMPs from various sources have been demonstrated to confer resistance against fungal and bacterial pathogens in an array of genetically engineered plant species, including *Arabidopsis* [21], tobacco [22]–[31], rice [32]–[37], potato [38]–[44], tomato [45], cotton [46], pear [47], banana [22], ornamental crops, geranium (*Pelargonium* sp.) [48], American elm [49] and hybrid poplar [50], [51].

Penaeidins, a family of AMPs originally isolated from the haemocytes of penaeid shrimp, is considered to be a source of compoundsthat have the potential to be applied in agriculture to deliver disease resistance to plants. Unlike vertebrates possessing the adaptive immune system, shrimp only have an innate immune system, among which are penaeidin antimicrobial peptides [52], [53]. Upon pathogen challenge to the host, the peptides are released from granular haemocytes to the plasma and attached to cuticles fighting microbial infection [53]–[56]. Penaeidins have a unique two-domain structure including an unconstrained proline-rich N-terminal domain (PRD) and a cysteine-rich domain (CRD) with a stable α-helical structure [53], [57], [58]. The complexity inherent in this unique structure might have contributed to its broad range of microbial targets, including primarily Gram positive bacteria and fungi [53], [54], [58], [59].

The penaeidin family is divided into four classes, designated as 2, 3, 4 and 5. Each class displays a remarkable level of primary sequence diversity [52], [60]. Pen4-1, an isoform within the class number 4 penaeidins (isoform number 1) is isolated from Atlantic white shrimp (*Litopenaeus setiferus*). It contains 6 cysteine residues forming 3 disulfide bridges and is the shortest isoform in penaeidin family with a length of 47 amino acids. It can inhibit multiple plant pathogenic fungal species, including the *B. cinera, P. crustosum* and *F. oxysporum* [53]. It is also effective against Gram-positive bacteria species including *M. luteus* and *A. viriduans*, and inhibitory against Gram-negative bacteria, *E. coli*, at relatively high concentrations [52]. Notably, Pen4-1 can inhibit the growth of multidrug-resistant fungi species: *Cryptococcus neoforman* (*Steroform A, Steroform B, Steroform C, Steroform D*) and *Candida spp.* (*Candida lipolytica, Candida inconspicua, Candida krusei, Candida lusitaniae* and *Candida glabrata*) [52]. Compared to other classes of penaeidins, penaeidin class 4 has shown a high level of potency against fungi [52]. Additionally, the unusual amino acid composition of Pen4-1, especially in the proline-rich domain, may confer resistance to proteases [52]. These results suggest that Pen4-1 is a good candidate for genetic engineering of enhanced disease resistance in plants. The present study investigates the feasibility of using the plant-optimized nucleotide sequences encoding Pen4-1 from *L. setiferus* for engineering fungal pathogen resistance into perennial turfgrass plants. We report the development of transgenic lines of a commercial creeping bentgrass (*Agrostis stolonifera* L.) cultivar, cv. Penn A-4 with enhanced resistance to two important fungal pathogens, *Sclerotinia homoecarpa* and *Rhizoctonia solani* as a result of expression of a synthetic peptide gene, *Pen4-1*.

## Results

### Production and molecular characterization of transgenic creeping bentgrass plants harboring the *Pen4-1* gene

**Figure 1:**
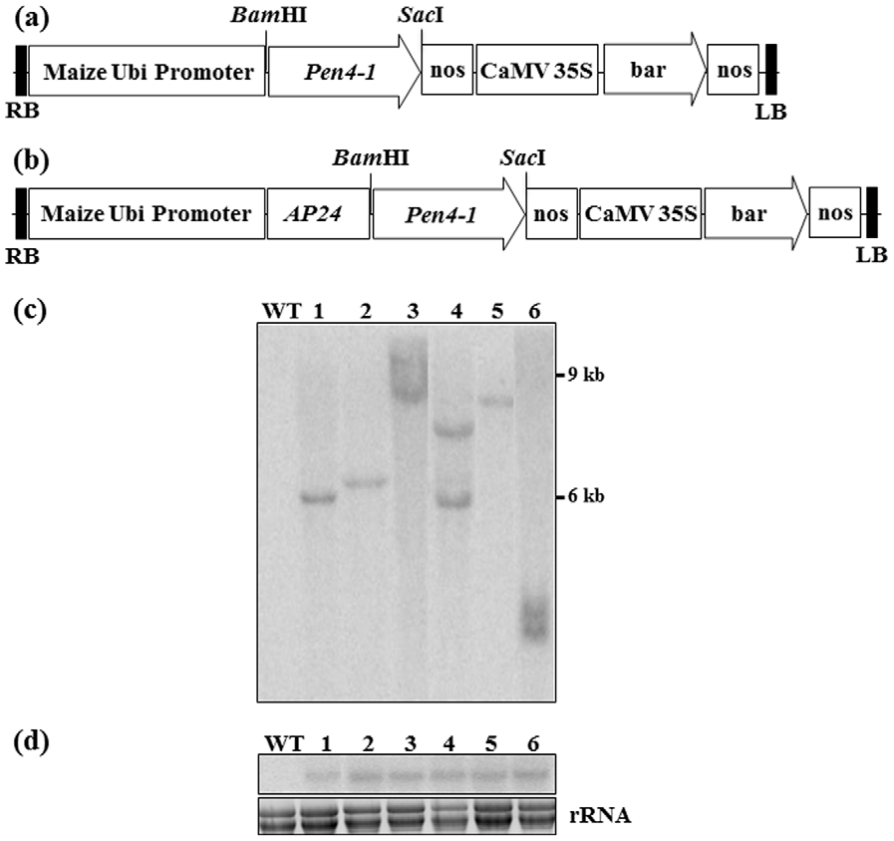
Generation and molecular analysis of the transgenic lines expressing *Pen4-1.* (a) Schematic diagram of the *Pen4-1* expression chimeric gene construct, p35S*-bar*/Ubi-*Pen4-1*. *Pen4-1* gene is under the control of the maize ubiquitin promoter (Ubi) and linked to the herbicide resistance gene, *bar*, driven by the CaMV 35S promoter. (b) Schematic diagram of the *AP24::Pen4-1* expression chimeric gene construct, p35S*-bar*/Ubi-*AP24::Pen4-1*, in which the *AP24::Pen4-1* fusion gene is under the control of the maize Ubi promoter. The CaMV35S promoter-driven *bar* gene is included for herbicide resistance. (c) Example of Southern blot analysis of *Pen4-1* expression transgenics. Twenty micrograms of the genomic DNA extracted from young leaves and digested with *Bam*HI that cuts once within the T-DNA region was probed by a 440 bp ^32^P-labelled *bar* gene fragment. Hybridization signals revealed were indication of copy numbers of transgene insertion. Lanes 1–6 were DNAs from representative transgenic creeping bentgrass plants. The negative control (WT) was *Bam*HI-digested genomic DNA from a non-transformed wild-type plant. (d) Example of Northern blot analysis of *Pen4-1* expression transgenics. Lanes 1-6 were total RNA from the same representative transgenic creeping bentgrass plants used for Southern analysis in (c). Twenty micrograms of the total RNA extracted from young leaves and probed with a ^32^P-labelled *Pen4-1* gene fragment. The negative control (WT) was total RNA from a non-transformed wild-type plant.

**Figure 2:**
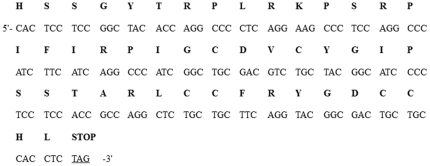
Nucleotide and deduced amino acid sequences of the *Pen4-1* gene. The original nucleotide sequences of *Pen4-1* were modified for plant-optimized codon usage. The predicted single-letter amino acids are shown above the coding sequence. The added translation stop codon is also indicated.

To generate transgenic plants expressing Pen4-1 and study the role Pen4-1 plays in plant disease resistance, two chimeric DNA constructs were prepared containing either the coding sequence of a single peptide Pen4-1 (Figure 1a) or the DNA sequence coding for the transit signal peptide of the secreted tobacco AP24 protein translationally fused to Pen4-1 coding sequence (Figure 1b). A maize ubiquitin (*ubi*) promoter was used in both constructs to drive *Pen4-1* expression and an herbicide resistance conferring gene named bar driven by the CaMV 35S promoter was included as a selectable marker for plant transformation. The original nucleotide sequences of *Pen4-1* were modified for plant-optimized codon usage, and chemically synthesized for use in chimeric gene construction (Figure 2).

Using *Agrobacterium-*mediated transformation of embryogenic callus derived from mature seeds and phosphinothricin selection, we separately introduced the two chimeric gene constructs (Figure 1a, 1b) into a creeping bentgrass (*A. stolonifera* L.) cultivar, Penn A-4, producing a total of 25 independent T_0_ transgenic lines transformed with the construct, p35S*-bar*/Ubi-*Pen4-1*, and 5 with the construct, p35S*-bar*/Ubi-*AP24::Pen4-1*. PCR amplification of foreign genes using genomic DNA from transgenic plants confirmed the presence of transgenes (data not shown). Southern hybridization with a *bar*-specific probe revealed that all the transgenic events contained one or two copies of transgene integration, most of which carried single copy insertions (see example in Figure 1c). No significant difference in general plant morphology, root and shoot development as well as overall plant biomass was observed between transgenic and control plants without *Pen4-1* gene.

### *Pen4-1* expression in transgenic plants of creeping bentgrass

Transgenic plants were further analyzed for *Pen4-1* expression by Northern blot analysis. Hybridization of RNA samples from leaves revealed detectable *Pen4-1* transcript, indicating transgene expression in all the transgenic plants (see examples in Figure 1d). Moreover, all transgenic lines, regardless of *Pen4-1* alone or *AP24::Pen4-1* fusion gene being expressed in plants, did not appear to show significant differences from each other for *Pen4-1* mRNA accumulation (data not shown). Our efforts in detecting Pen4-1 protein in plant extracts from turfgrass transgenic lines using a polyclonal antibody raised against the selected region of Pen4-1 protein was unsuccessful (data not shown). This difficulty in detecting Pen4-1 protein in turfgrass plants was also encountered when analyzing Pen4-1 production in *Arabidopsis* transgenic lines expressing the *Pen4-1* gene (data not shown). Many attempts in improving protein extraction and immunoblotting using currently available methodology and published procedures did not result in satisfactory results. This difficulty in Western assay with Pen4-1 may result from poor retention of the protein by blotting membranes due to its small size and a highly positive charge. Protease degradation of Pen4-1 during protein extraction could be another possibility, but is unlikely given the unusual amino acid composition of PRD Pen4-1 conferring resistance to proteases [52]. The same problem had been reported previously for other plant-expressed small AMPs [27], [32], [42], [46].

Four representative transgenic lines harboring p35S*-bar*/Ubi-*Pen4-1* and 5 containing the construct, p35S*-bar*/Ubi-*AP24::Pen4-1* were used in the subsequent pathogen test experiments, all of which contained a single copy integration of the transgene. These transgenic lines were clonally multiplied by vegetative propagation. Evaluation of these plants under greenhouse conditions showed that they performed very similarly in growth. Three groups of control plants were used for comparison with the Pen4-1-expressing transgenic lines in our pathogen test experiments. They were untransformed plants either derived from seeds or regenerated from tissue culture and transgenic lines harboring the same expression vector without the *Pen4-1*, but with a different foreign gene, which was not associated with plant response to pathogen attack. All control plants, regardless of their origins, did not show significant differences in morphology and growth as well as response to pathogen infection.

### *In planta* antifungal assays with *R. solani*

*R. solani* is a soil-borne fungus that causes brown patch disease, one of the most severe diseases on turfgrass lawns. To examine the impact of Pen4-1 on plant response to infection with *R. solani*, we conducted experiments investigating plant disease resistance by both *in vitro* and *in vivo* assays using detached leaves and whole plants, respectively.

**Figure 3:**
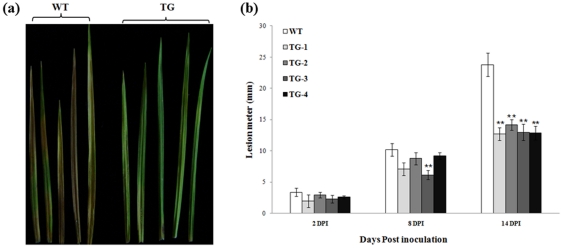
Response of transgenic creeping bentgrass plants expressing Pen4-1 to *R. solani* infection - in vitro plant leaf inoculation assay. (a) The detached second expanded leaves from the top of plant stolons were used for pathogen inoculation test. The image shows example of representative leaves from all tested Pen4-1-expressing transgenic plants with a single transgene insertion (TG, on the right) and wild-type control plants (WT, on the left) 14 days post-inoculation (DPI). Transgenic plants exhibited significant resistance to *R. solani* in comparison to wild-type controls. (b) The development of brown patch disease was rated by measuring the lesion length of the infected leaves 2, 8 and 14 DPI. Statistical analysis of *R. solani* inoculation test was conducted on wild-type control plants (WT) and various transgenic lines harboring either p35S*-bar*/Ubi-*Pen4-1* (TG1 and TG2) or p35S*-bar*/Ubi-*AP24::Pen4-1* (TG3 and TG4). Data are presented as means ± SE (n = 10), and error bars represent standard error. Asterisks (** or *) indicate a significant difference between Pen4-1-expressing transgenic and control plants at *P*<0.01 or *P*<0.05 by Tukey's HSD test using JMP 9.0.0. The *P* values are listed in Table S1.

The detached leaves from T_0_ transgenic lines expressing Pen4-1 and the control plants were placed on 1% of agar in Petri dishes, and challenged by the pathogen using agar plugs infested with mycelium of *R. solani* isolate obtained from infected creeping bentgrass plants. Disease symptoms measured as lesion size were documented at various times after inoculation. Compared to control plants, transgenic lines harboring either p35S*-bar*/Ubi-*Pen4-1* or p35S*-bar*/Ubi-*AP24::Pen4-1* exhibited dramatically enhanced disease resistance with a reduction in lesion length by 42% to 48% fourteen days after inoculation (Figure 3a, b). Statistical analysis of Tukey's Hornesly Significant Difference (Tukey's HSD) indicated that the lesion size reduction in Pen4-1-expressing transgenic plants was significant (*P*<0.01); whereas, no significant difference in lesion size was observed among Pen4-1-expressing transgenic lines (*P*>0.05) (Figure 3b).

**Figure 4:**
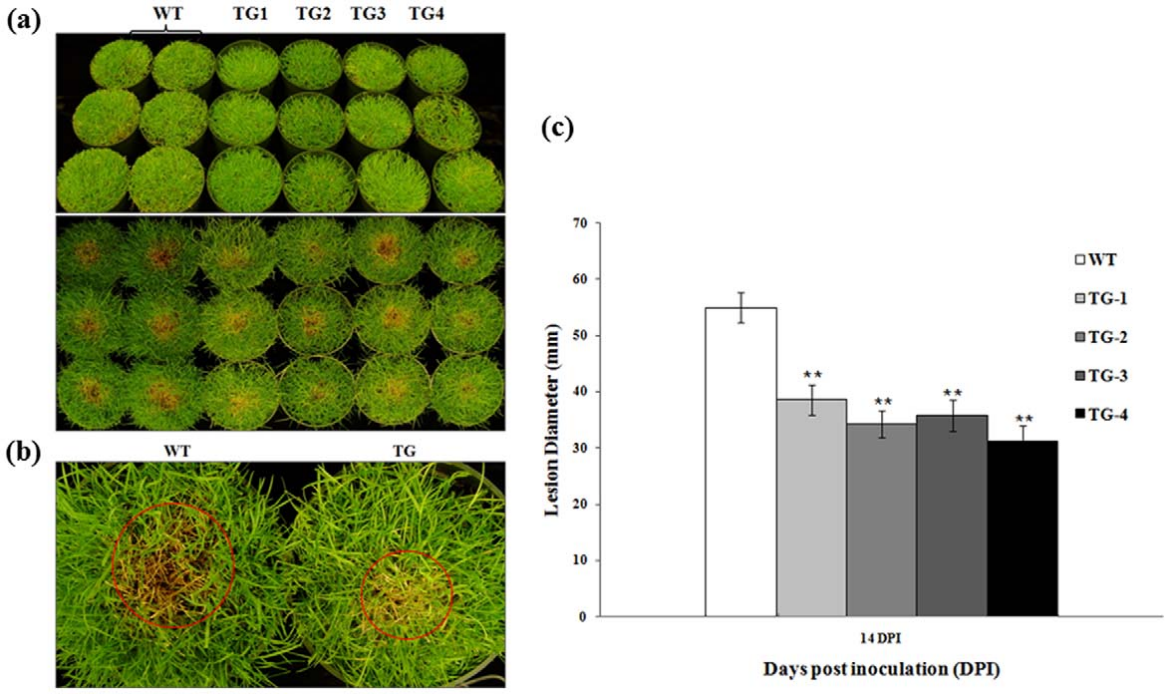
Response of transgenic creeping bentgrass plants expressing Pen 4-1 to *R. solani* infection - in vivo direct plant inoculation bioassays with lower dose of *R. solani*. (a) The fully developed transgenic (independent events TG1 to TG4) and wild-type (WT) plants clonally propagated from individual stolons were grown and maintained in pots (15 cm×10.5 cm) and inoculated with 3 g of rye seeds colonized by *R. solani.* The image on the upper panel shows plants before pathogen infection. Example of plants from wild-type (WT) and representative transgenic lines harboring either p35S*-bar*/Ubi-*Pen4-1* (TG1, TG2) or p35S*-bar*/Ubi-*AP24::Pen4-1* (TG3 and TG4) two weeks after pathogen inoculation (14 DPI) are shown on the bottom panel. Transgenic plants exhibited less sever disease symptom than wild-type controls. (b) A closer look of infected plants showing the different lesion size of WT and TG. (c) The development of brown patch disease was rated by measuring the lesion diameters of the infected leaves 14 DPI. Statistical analysis of *R. solani* inoculation test was conducted on WT and various TG lines. Data are presented as means ± SE (n = 6), and error bars represent standard error. Asterisks (** or *) indicate a significant difference between transgenic plants and wild-type controls at *P*<0.01 or *P*<0.05 by Wilcoxon test using JMP 9.0.0. The *P* values are listed in Table S2.

Plant performance in response to an *R. solani* infection was further evaluated by *in vivo* assays using the whole plants grown in pots. Control plants without Pen4-1 and transgenic lines harboring either p35S*-bar*/Ubi-*Pen4-1* or p35S*-bar*/Ubi-*AP24::Pen4-1* were both challenged by the pathogen in replicated experiments under a controlled environment. Plants in each pot were inoculated with 3 grams of rye seeds colonized by *R. solani*. Pen4-1-expressing transgenic lines all exhibited high resistance against pathogen infection with a reduced lesion diameter by 30% - 43% compared to control plants 14 days after inoculation (Figure 4a, b and c). Statistical analysis of Wilcoxon test indicated that the disease symptoms among the different Pen4-1-expressing transgenic lines were not significant (*P*>0.05) (Figure 4c), whereas a significant difference in disease development between control and Pen4-1-expressing transgenic plants was observed (*P*<0.01) (Figure 4c).

**Figure 5:**
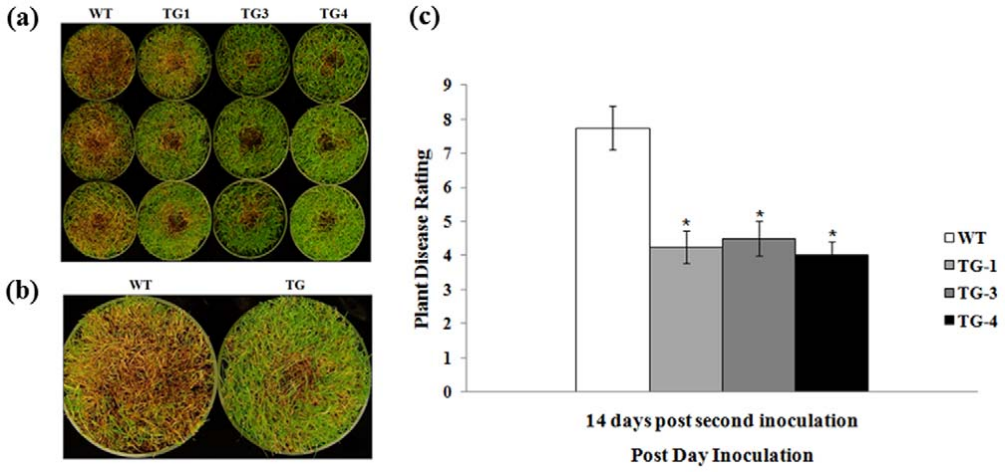
Response of transgenic creeping bentgrass plants expressing Pen 4-1 to *R. solani* infection - in vivo direct plant inoculation bioassays with higher dose of *R. solani*. (a) Transgenic (TG) and wild-type (WT) plants were inoculated with a second dose of *R. solani* (3g of rye seeds colonized by the pathogen) 14 days after the first inoculation with 3 g of rye seeds colonized by *R. solani.* The image shows example of plants from wild-type (WT) and representative transgenic lines harboring either p35S*-bar*/Ubi-*Pen4-1* (TG1) or p35S*-bar*/Ubi-*AP24::Pen4-1* (TG3 and TG4) two weeks after the second pathogen inoculation. Transgenic plants exhibited much less sever disease symptom than wild-type controls. (b) A closer look of infected plants showing the different lesion size of WT and TG. (c) The development of brown patch disease was rated by visual estimation of the lesion percentage of the infected leaves 14 DPI using the Horsfall/Barrett scale. Statistical analysis of *R. solani* inoculation test was conducted on WT and various TG lines. Data are presented as means ± SE (n = 6), and error bars represent standard error. Asterisks (** or *) indicate a significant difference between transgenic plants and wild-type controls at *P*<0.01 or *P*<0.05 by Wilcoxon test JMP 9.0.0. The *P* values are listed in Table S3.

When exposed to a second dose of pathogen infection, *i.e.* plants in each pot were inoculated with additional 3 grams of rye seeds colonized by *R. solani* 14 days after the first inoculation, the control plants suffered severe damage with 75% to 95% of them in the pots being affected two weeks after inoculation, whereas Pen4-1-expressing transgenic lines were much less impacted with only around 25% of plants in the pots being infected (Figure 5a, b). The disease ratings of the Pen4-1-expressing transgenic lines were reduced by 41% to 44% compared to that of the control plants (Figure 5c). Statistical analysis of Wilcoxon test indicated that the disease development among the different Pen4-1-expressing transgenic lines was not significant (*P*>0.05) (Figure 5c).

### *In planta* antifungal assays with *S. homoeocarpa*

Transgenic plants expressing Pen4-1 were also evaluated for their resistance to dollar spot, another important turfgrass disease caused by *S. homoeocarpa* [61], [62]. Both *in vitro* and *in vivo* assays were conducted to examine the impact of Pen4-1 on plant response to infection with *S. homoeocarpa*.

**Figure 6:**
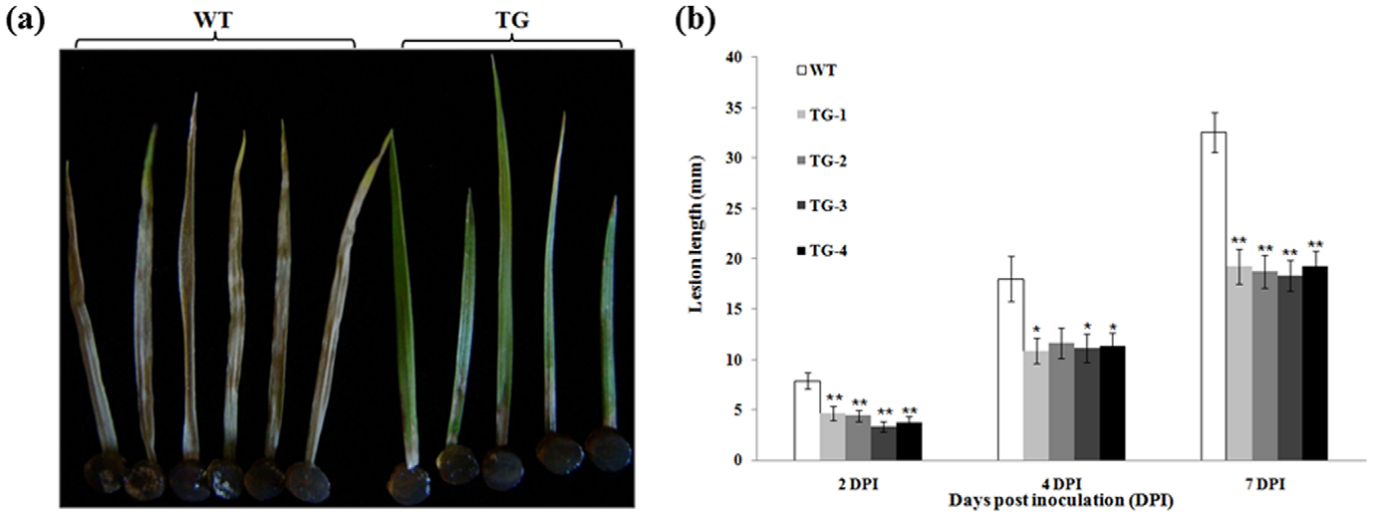
Response of transgenic creeping bentgrass plants expressing Pen 4-1 to *S. homoeocarpa* infection - in vitro plant leaf inoculation assay. (a) The detached second expanded leaves from the top of plant stolons were used for pathogen inoculation test. The image shows example of representative leaves from all tested Pen 4-1-expressing transgenic plants with a single transgene insertion (TG, on the right) and wild-type control plants (WT, on the left) 7 days post-inoculation (DPI). Transgenic plants exhibited significant resistance to *S. homoeocarpa* in comparison to wild-type controls. (b) The development of dollar spot disease was rated by measuring the lesion length of the infected leaves 2, 4 and 7 DPI. Significant resistance to *S. homoeocarpa* by transgenic plants was observed 7 DPI when compared to wild-type controls. Statistical analysis of *S. homoeocarpa* inoculation test was conducted on wild-type control plants (WT) and various transgenic lines harboring either p35S*-bar*/Ubi-*Pen4-1* (TG1 and TG2) or p35S*-bar*/Ubi-*AP24::Pen4-1* (TG3 and TG4). Data are presented as means ± SE (n = 10), and error bars represent standard error. Asterisks (** or *) indicate a significant difference between transgenic plants and wild-type controls at *P*<0.01 or *P*<0.05 by Tukey's HSD test using JMP 9.0.0. The *P* values are listed in Table S4.

*In vitro* assays were conducted using leaves from T_0_ Pen4-1-expressing transgenic lines and control plants. The detached leaves were placed on 1% of agar in Petri dishes, and challenged by the pathogen using *S. homoeocarpa*-infested agar plugs. Disease symptoms measured as lesion size were documented at various times after inoculation. Compared to control plants, transgenic lines harboring either p35S*-bar*/Ubi-*Pen4-1* or p35S*-bar*/Ubi-*AP24::Pen4-1* all exhibited dramatically enhanced disease resistance with a reduction in lesion length by 40% to 47% seven days after inoculation (Figure 6a, b). Statistical analysis of Tukey's HSD indicated that the lesion size reduction in Pen4-1-expressing transgenic lines was significant (*P*<0.05), whereas no significant difference in lesion size was observed among Pen 4-1-expressing transgenic lines (*P*>0.05) (Figure 6b).

**Figure 7:**
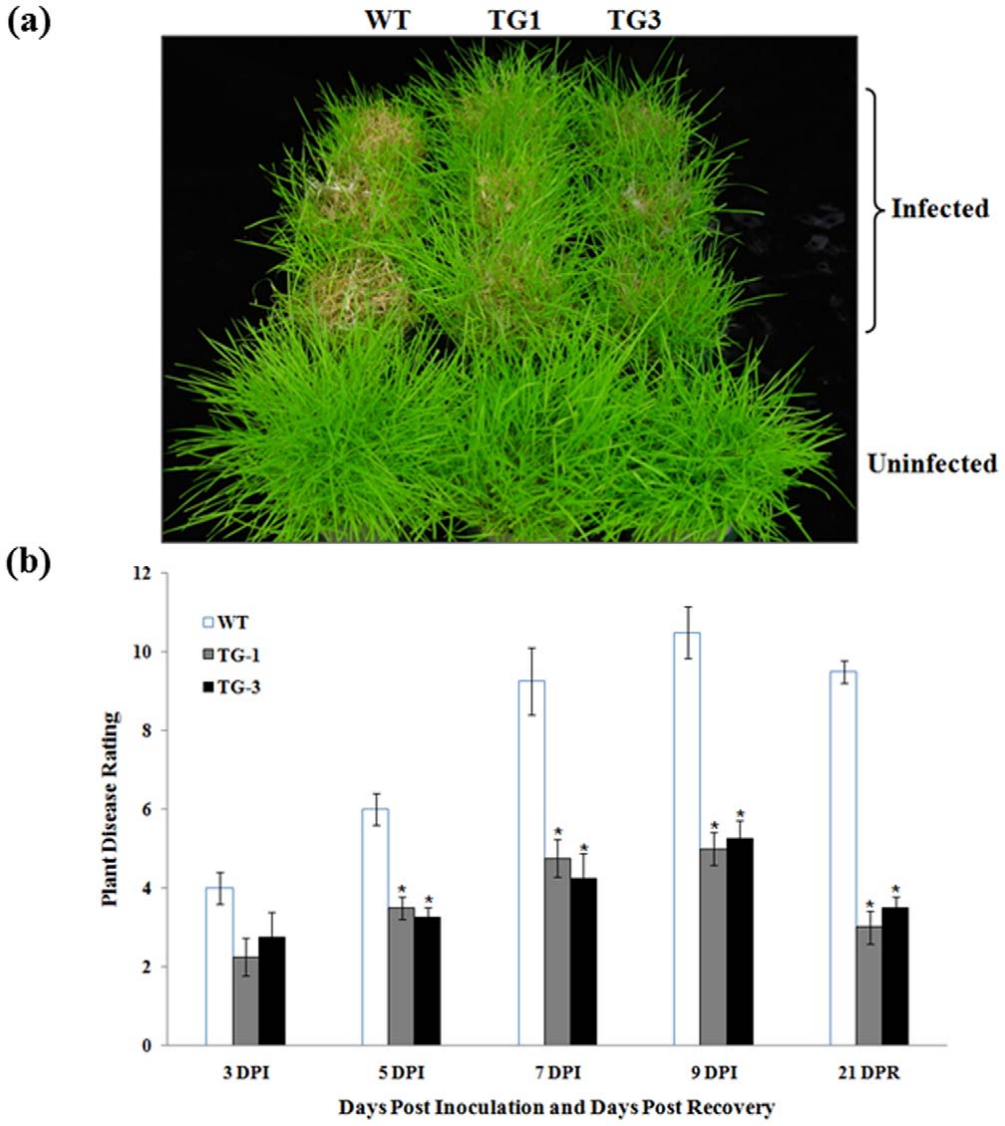
Response of transgenic creeping bentgrass plants expressing Pen 4-1 to *S. homoeocarpa* infection - in vivo direct plant inoculation bioassays with higher dose of *S. homoeocarpa*. (a) The fully developed transgenic (TG) and wild-type (WT) plants clonally propagated from individual stolons were grown and maintained in pots (15 cm×10.5 cm) and inoculated with 0.5 g of rye seeds colonized by *S. homoeocarpa.* The image shows example of plants from wild-type (WT) and representative transgenic lines harboring either p35S*-bar*/Ubi-*Pen4-1* (TG1) or p35S*-bar*/Ubi-*AP24::Pen4-1* (TG3) 9 days after pathogen inoculation (9 DPI). The plants in the front row are uninfected controls. Transgenic plants exhibited significant disease resistance compared to wild-type controls. (b) The development of dollar spot disease was rated by visual estimation of the lesion percentage of the infected leaves 3, 5, 7, 9 DPI, and 21 days post-recovery (DPR) using the Horsfall/Barrett scale. Statistical analysis of *S. homoeocarpa* inoculation test was conducted on WT and various TG lines. Data are presented as means ± SE (n = 6), and error bars represent standard error. Asterisks (** or *) indicate a significant difference between transgenic plants and wild-type controls at *P*<0.01 or *P*<0.05 by Wilcoxon test using JMP 9.0.0. The *P* values are listed in Table S5.

Plant performance in response to *S. homoeocarpa* infection was further evaluated by *in vivo* assays using the whole plants grown in big pots. Control plants and transgenics harboring either p35S*-bar*/Ubi-*Pen4-1* or p35S*-bar*/Ubi-*AP24::Pen4-1* were both challenged by the pathogen in replicated experiments under a controlled environment. Pen4-1-expressing transgenic lines all exhibited high resistance against pathogen infection with disease ratings reduced more than 50% compared to various control plants 9 days after inoculation (Figure 7a, b). Statistical analysis of the Wilcoxon test indicated that disease development in all the Pen4-1-expressing transgenic lines was significantly delayed (Figure 7b) and in the recovery phase, transgenic lines performed much better than control plants (*P*<0.05). However, no significant difference in disease resistance among Pen4-1-expressing transgenic lines was observed (*P*>0.05) (Figure 7b).

## Discussion

The results reported herein show that Pen4-1, one of the penaeidin proteins isolated from Atlantic white shrimp (*Litopenaeus setiferus*), when expressed in transgenic perennial grass plants, confers antifungal traits. Transgenic creeping bentgrass plants expressing Pen4-1 exhibited significantly enhanced resistance to dollar spot and brown patch, the two major fungal diseases in turfgrass caused by *S. homoecarpa* and *R. solani* respectively. To our knowledge, this is the first report of genetically engineering an economically and environmentally important perennial grass species with a gene encoding an AMP from the class four isoform of the shrimp penaeidin family for enhanced resistance against two fungal pathogens. There was only one recent study reporting the use of penaeidin protein for plant disease resistance [63]. In that study, Np3 and Np5, the two AMPs belonging to class 3 and 5 of the penaeidin family from Chinese shrimp (*Fenneropenaeus chinensis*) [64], [65] were engineered into rice and the four transgenic lines generated were reported to show enhanced resistance to bacterial blight (*Xanthomonas oryzae*).

In the present study, the *Pen4-1* gene with plant-preferred codon usage was chemically synthesized for chimeric gene construction and plant transformation. The 30 transgenic turfgrass lines constitutively expressing either the *Pen4-1* gene (25) or the *AP24::Pen4-1* fusion gene (5) all contained one or two copies of the integrated transgene and were normal in morphology and development. Pen4-1 expression was confirmed at the transcription level (Figure 1d). *In planta* disease resistance assays to compare transgenic lines expressing Pen4-1and control plants without Pen4-1 for their response to two important turfgrass fungal pathogens clearly demonstrated the effectiveness of this novel AMP in rendering transgenic plants with significantly enhanced resistance to both brown patch and dollar spot diseases. It is unlikely that the observed results would be attributed to disrupted genes or regulatory sequences at the transgene integration site(s) since Pen4-1-expressing transgenic lines from independent transformation events all show similar phenotypes and confer increased resistance to fungal pathogens, whereas transgenic control plants that contain the same expression vector, but without *Pen4-1*, do not exhibit enhanced performance when subjected to pathogen infection. It also should be noted that in the current research, T_0_ transgenic plants were clonally propagated and used for pathogenicity assays as previously reported in other studies on perennial grasses [66]. Our earlier work studying transgene expression and transmission using the selectable marker, herbicide resistance conferring gene *bar* in creeping bentgrass had demonstrated that *Agrobacterium*-mediated transformation of creeping bentgrass led to a high frequency of a single-copy transgene insertion that exhibited stable inheritance patterns. The inheritance and stability of transgene were demonstrated in both greenhouse and field conditions [67]–[70]. Currently, we are also conducting experiments studying stable transmission of *Pen4-1* into next generations and inheritance of the enhanced disease resistance trait by the progeny of the primary transgenic plants. Data from this research will provide further support facilitating large-scale application of Pen4-1 in turf species for plant protection.

Since Pen4-1 originates from shrimp, the successful use of this protein in agricultural biotechnology requires that it be efficiently produced when *Pen4-1* gene is introduced into the plant host genome, and that its biological activity maintained when produced in transgenic plants. Although the antifungal activity of Pen4-1 has been demonstrated by *in vitro* test of the synthesized protein [52], [53], [57], it remains to be determined whether or not high-level gene expression, efficient protein production, and correct folding or processing of this protein could be achieved *in planta*. We therefore modified the coding sequence of Pen4-1 for monocot plant-preferred codon usage. The same strategy has been used previously when introducing other non-plant-derived AMP genes in plants [32]. In our study, a RNA transcript of the codon-optimized *Pen4-1* gene was detected in all the transgenic lines. Although attempts in detecting protein products of the *Pen4-1* in transgenic lines were unsuccessful, transgenic plants all displayed significantly enhanced resistance to the two major turfgrass fungal diseases compared to wild-type controls indicating that Pen4-1 was successfully produced in transformed creeping bentgrass plants. It should be noted that native AMP genes have also been reported to be expressed and function in other plant systems. For example, when introducing *Aspergillus giganteus* antifungal protein AFP into rice plants, the translational efficiencies of transcripts originating from the native and codon-optimized *AFP* gene appeared similar in transgenic plants [32]. Similarly, the introduction of *np3* and *np5*, two other AMP genes from Chinese shrimp in their native forms into rice led to enhanced plant resistance to bacterial blight. This suggested the production of active AMPs in transgenic plants although the presence of the protein products in transgenic plants was not demonstrated [63]. Further transgenic studies to compare the original and codon-optimized forms of *Pen4-1* gene for their protein translation efficiencies would facilitate its use in other plant systems to achieve enhanced disease resistance.

All penaeidins possess a unique two-domain structure including an unconstrained proline-rich N-terminal domain, PRD and a disulfide bond-stabilized cysteine-rich domain, CRD [57]. To ensure efficient disulfide bond formation of the Pen4-1 produced in transgenic creeping bentgrass plants, we prepared a chimeric gene encoding a fusion protein in which the DNA sequence coding for the transit signal peptide of the secreted tobacco AP24 protein was translationally fused to the Pen4-1 coding sequence. Transit signal peptides, such as the one from AP24, are known to be capable of directing proteins into the endoplasmic reticulum, facilitating the formation of disulfide bonds [32]. Therefore, the AP24::Pen4-1 fusion protein produced in plant cells should lead to mature Pen4-1 that is more likely to be correctly folded than the Pen4-1 protein alone produced in transgenic plants. However, in the present study, we did not observe dramatic differences in enhanced plant resistance to the two turfgrass fungal pathogens between transgenic turfgrass lines expressing *AP24::Pen4-1* fusion gene and those expressing *Pen4-1* gene alone. One possible explanation could be that a minimal protein activity was enough to inhibit pathogen infection; therefore, the methods for pathogenicity assays used in the present study could not detect the real difference in protein activities between the Pen4-1 alone and the AP24::Pen4-1 fusion protein expressed in transgenic plants. Another possibility would be that although the mechanism of protein secretion is highly conserved through the living world [71], signal peptides from one organism do not always function efficiently when expressed in another organism [71]–[73]. The correct choice of the signal peptide would have a great effect on the production of the AMPs.

The multi-domain structure and the feature of protease resistance of this peptide may also play important role in bestowing more flexibility to protein processing and determining protein activities [52]. In most cases the presence of both the CRD and the PRD are important to confer the maximal antimicrobial activity. However, it has been demonstrated that the single Pen4 PRD alone exhibited a similar level of antimicrobial activity to that of the full-length Pen4 [53], [57]. This implies that the disulfide bond formation in Pen4 may not play a critical role in its antimicrobial ability. Therefore, specific targeting of Pen4-1 to endoplasmic reticulum by the AP24 signal peptide did not seem to result in enhanced protein activity. It is also possible that pathogen attack would lead to disruption of the plant cells, releasing the peptides, which could then be oxidized in the extracellular space to form the disulfide bond. Further studies comparing Pen4 PRD alone and the full-length Pen4 in transgenic plants for their antimicrobial activities would help better understand the role disulfide bonds play in determining the overall activity of Pen4 proteins.

The *in vitro* tests of Pen4-1 have revealed its resistance to a wide range of phytopathogens, of which many infect plant species including rice, wheat, wine grapes, strawberry and other crop plants [52]. The current study with creeping bentgrass as a target species provides the very first example of using Pen4-1 for genetic engineering of enhanced disease resistance in transgenic crop plants, pointing to the great potential of implementing similar strategies in other plant systems, especially in food crops for improvement of plant biotic stress resistance.

## Materials and Methods

### Synthesis of *Pen4-1* gene

The full sequence of *Pen4-1* gene was obtained from PenBase [74]. The original nucleotide sequences of *Pen4-1* encoding the mature Pen4-1 protein (47 amino acids) were modified for plant-optimized codon usage. A stop codon (TAG) was added to the 3' end of the coding sequence (Figure 2). The modified full sequence of *Pen4-1* was chemically synthesized by Integrated DNA Technology (Coralville, IA, USA), cloned in pZErO-2 (Invitrogen, Carlsbad, CA, USA) and verified by sequencing.

### Construction of plant expression vectors

To generate transgenic plants expressing Pen4-1 and study the role Pen4-1 plays in plant disease resistance, two chimeric DNA constructs were prepared containing either the coding sequence of a single peptide Pen4-1 (Figure 1a) or the DNA sequence coding for the transit signal peptide of the secreted tobacco AP24 protein translationally fused to Pen4-1 coding sequence (Figure 1b). Both constructs were prepared using a pSB11-based *Agrobacterium* binary vector that contains a selectable marker gene conferring antibiotic spectinomycin resistance for bacterial transformation [75].

The two plant expression vectors, p35S*-bar*/Ubi-*Pen4-1*, and p35S*-bar*/Ubi-*AP24::Pen4-1* constructed in this work are presented in Figures 1a and b. Plasmid p35S*-bar*/Ubi-*Pen4-1* (Figure 1a) contained only the single peptide sequence of the codon-optimized *Pen4-1* gene, whereas plasmid p35S*-bar*/Ubi-*AP24::Pen4-1* (Figure 1b) contained a chimeric *Pen4-1* gene with the DNA sequence coding for the transit signal peptide of the secreted tobacco AP24 protein [76] being translationally fused to Pen4-1 coding sequence. For their expression in turfgrass, both *Pen4-1* and *AP24::Pen4-1* were cloned between the maize *ubi* promoter and the *nos* terminator. An herbicide resistance conferring gene named *bar* driven by the CaMV 35S promoter was included in both plasmids as selectable marker for plant transformation.

To prepare p35S*-bar*/Ubi-*Pen4-1*, the synthesized *Pen4-1* coding sequence (with added stop codon) was PCR amplified from pZErO-2:*Pen4-1* by primers Pen4-ATG: 5′-CGCGGATCC *ATG*CACTCCTCCGGCTACACC-3′ (a *Bam*HI restriction site and a start codon, ATG added in the 5′ end were underlined and in italic respectively) and Pen4R: 5′-CGCGCATGCGAGCTCTAGAGGTGGCAGCAGTCG-3′ (an *Sph*I and an *Sac*I restriction sites added in the 5′ end were underlined). The amplified fragment was digested with *Bam*HI and *Sac*I enzymes and ligated into the corresponding sites of p35S-*bar/Ubi-GUS* (Luo, unpublished results) to replace the *gusA* coding sequence. To prepare p35S*-bar*/Ubi-*AP24::Pen4-1* construct, the PCR amplified fragment of *Pen4-1* using Pen4F (5′-CACTCCTCCGGCTACACC-3′) and Pen4R primers was treated with DNA polymerase I, large (Klenow) fragment (New England Biolabs, Beverly, MA, USA) in the absence of dNTP, then digested with *Sph*I and ligated into the *Nco*I (blunt-ended with Klenow in the presence of dNTP)-*Sph*I sites of the plasmid pGEM-T-*AP24* [32], resulting in pGEM-T-*AP24::Pen4-1* (data not shown). Upon verification of the correct sequence of the amplified *Pen4-1* and its in-frame fusion to the *AP24* signal sequence, the *AP24::Pen4-1* chimeric gene was released from pGEM-T-*AP24::Pen4-1* by *Bam*HI and *Sac*I digestions and ligated into the corresponding sites of p35S-*bar/Ubi-GUS* to replace the *gusA* coding sequence. The two constructs were transformed into *Agrobacterium tumefaciens* strain LBA4404 by electroporation for subsequent plant transformation.

### Production, propagation and maintenance of transgenic turfgrass plants

A commercial genotype of creeping bentgrass (*A. stolonifera* L.) Penn A-4 was used for plant transformation. Transgenic creeping bentgrass lines harboring either p35S*-bar*/Ubi-*Pen4-1* or p35S*-bar*/Ubi-*AP24::Pen4-1* were produced by *Agrobacterium*-mediated transformation of embryonic callus initiated from mature seeds essentially as previously described [67]. Transgenic plants were grown in commercial potting mixture soil (Fafard 3-B Mix, Fafard Inc., Anderson, SC, USA) and maintained in the greenhouse under a 16-hour photoperiod with supplemental lighting at 27°C in the light and 25°C in the dark. Plants from individual transformation events were clonally propagated from stolons and grown in pots (15 cm×10.5 cm, Dillen Products, Middlefield, OH, USA) using commercial potting mixture soil as previously described [70]. Propagated plants were maintained in greenhouse for 4 to 6 months with regular fertilization, mowing and irrigation, and used for further analysis.

### Plant DNA isolation and southern blot analysis

Plant genomic DNA was extracted as previously described using the cetyltrimethyl ammonium bromide (CTAB) method [69]. After digestion of the DNA with *Bam*HI according to supplier's instruction (New England Biolabs, Beverly, MA, USA), DNAs were electrophoresed on 0.8% agarose gels, transferred onto nylon membranes (GE Healthcare Bio-Sciences Corp., Piscataway, NJ, USA), and hybridized to ^32^P-labelled DNA probes of *bar*. Hybridization was carried out in modified Church and Gilbert buffer at 65°C following the standard protocol [77]. Hybridizing fragments were detected by exposure of the membrane on a phosphor screen at RT overnight, and scanning on a Typhoon 9400 phosphorimager.

### RNA isolation and northern blot analysis

Total RNA was isolated from the leaves of transgenic and wild-type control plants using Trizol reagent (Invitrogen). RNAs were subjected to formaldehyde-containing agarose gel electrophoresis, and transferred onto Hybond-N^+^ filters (GE Healthcare Bio-Sciences Corp.). The DNA fragment coding for the *Pen4-1* gene was used as probe. Hybridization and membrane wash were performed following the standard protocol [77].

### Western blot Analysis

Total proteins were extracted from leaves following two different procedures. The first was essentially as described by Fu *et al*. [78]. Two hundred mg of leaf tissue was ground, then suspended in 500 *µ*l of protein extraction buffer [1×PBS (pH 7.4), 10 mM EDTA, 1 mM PMSF, 6 *µ*l protease inhibitor cocktail for plant cell and tissue extracts (sigma), 1% (v/v) *β*-mercaptoethanol, and 0.1% (v/v) Triton X-100]. The second protocol was as described by Coca *et al.* [32]. Four hundred mg of plant material was ground in liquid nitrogen and homogenized in SDS PAGE loading buffer without 2-mercaptoethanol and incubated at 95°C for 10 min. After centrifugation, the supernatant was precipitated with 4 volumes of acetone at −20°C for 30 min. Proteins were pelleted, dried and dissolved in SDS-PAGE loading buffer containing 5% 2-mercaptoethanol. For protein analysis, 30 *µ*g of protein sample was loaded onto a 16% Tricine SDS-PAGE gel. SDS-PAGE was performed as described previously [79]. For western blot, protein was transferred from the SDS-PAGE gel onto Protran BA76 Nitrocellulose media (Whatman Inc., Piscataway, NJ, USA) using an electrophoresis blotting system (Bio-Rad, Hercules, CA, USA). The Protein transfer efficiency was verified by using Poceau S, and incubated with 5% carnation nonfat dry milk in TBST overnight. The blots were then probed with anti-Pen4-1 antibody developed by YenZym Antibodies, LLC (Burlingame, CA, USA), using the peptide HSSGYTRPLRKPSRC, followed by adding the HRP-conjugated goat anti-rabbit IgG secondary antibody (Jackson ImmunoResearch Laboratories, Inc., West Grove, PA, USA) and incubation for 1 hour at room temperature with shaking. The signals were detected by incubation of membrane for 30 minutes at room temperature in the substrate working solution (4-Chloro-1-naphthol). After stopping the reaction by rinsing the membrane with water, the membrane was photographed immediately.

### In vitro plant leaf inoculation with *R. solani* and *S. homoeocarpa*

Transgenic plants were challenged with *R. solani* and *S. homoeocarpa*, which respectively cause brown patch and dollar spot, the two most common fungal diseases in creeping bentgrass. Following procedures modified from the previous reports [43], [80]–[82], we grew the *R. solani* and *S. homoeocarpa* cultures on potato dextrose agar at 25°C for 3 days prior to inoculation of detached leaves under aseptic conditions. The second expanded leaves from the top of plant stolons were used for inoculation. Ten leaves from each of the transgenic lines and wild-type control plants were randomly chosen for study. The leaves cut from plants were first washed with 70% ethanol, and then rinsed with sterilized water. The leaves were put on 1% of agar in Petri dishes (150×15 mm). An agar plug (d = 3 mm) infested with mycelium of *R. solani* or *S. homoeocarpa* was placed on the bottom of the midrib of each detached leaf for inoculation. The Petri dishes were put in a lighted growth chamber under a 14/10 h (day/night) photoperiod. Temperature and relative humidity (RH) in the growth chamber were 28°C and 70%. The development of brown patch disease was rated by measuring the lesion length of the infected leaves 2 days, 8 days and 14 days post-inoculation. The development of dollar spot disease was rated by measuring the lesion length on the infected leaves 2 days, 4 days and 7 days post-inoculation. The experiment was repeated three times.

### In vivo direct plant inoculation with *S. homoeocarpa* and *R. solani*

The preparation of the *S. homoeocarpa* and *R. solani* cultures and the *in vivo* plant inoculation with pathogens were conducted based on the previously reported procedures [3], [83], [84]. Selected Pen4-1-expressing transgenic lines based on molecular analysis were evaluated for resistance to the infection of the two fungal pathogens in comparison to control plants that did not contain Pen4-1. The grasses were mowed prior to inoculation. The plants in each pot were then inoculated with pathogens by applying, in the center of the pot, approximately 0.5 g of rye seeds colonized by *S. homoeocarpa* or 3 and 6 g of rye seeds colonized by *R. solani.*

Plants inoculated with *S. homoeocarpa* (0.5 g of colonized inoculum) were placed in plastic trays containing 4 cm of distilled water, lightly misted with distilled water at 48 h intervals to maintain relative 100% humidity. The plastic trays were placed inside a greenhouse set to maintain a diurnal cycle of 14 h light and 10 h dark. Three to four replicates of each transgenic line or wild type control were used for evaluation. Disease severity was visually estimated at 3, 5, 7 and 9 days post-inoculation using the Horsfall/Barrett scale [85]. Nine days later, the plants were moved to a growth room from the greenhouse to recover for three weeks. Temperatures in the growth room were maintained at 22°C in the light and 17°C in the dark. The inoculation experiment was repeated three times.

Plants inoculated with *R. solani* (3 g rye grass seeds colonized by the pathogen) were placed in plastic trays containing 4 cm of distilled water, lightly misted with distilled water at 48 h intervals to maintain humidity. The trays were placed inside a growth chamber to maintain a diurnal cycle of 14 h light and 10 h dark. The temperature and RH were 30°C and 70% during day time, and 24°C and 95% at night. After 14 days, disease severity was either rated by measuring the total distance from the point of inoculation to the farthest point of the lesions extended, or visually estimated using the Horsfall/Barrett scale [85]. The inoculation experiment was repeated twice.

### Statistical analysis

Both *in vitro* plant leaf inoculation and *in vivo* direct plant inoculation tests were conducted using a randomized complete block design. Data were analyzed using JMP® 9.0.0 (2010 SAS Institute Inc). For the data generated using the Horsfall-Barratt scale, a nonparametric test, Wilcoxon test at *P* = 0.01 and *P* = 0.05, was used to compare the medians. In the case of the data generated on a continuous scale such as lesion length, Tukey's HSD at *P* = 0.01 and *P* = 0.05 was used to test for differences in mean disease severity.

## Supporting Information

Table S1 ***P*** **values of in vitro plant leaf inoculation assay with** ***R. solani*** **.**
(DOCX)

Table S2 ***P*** **values of in vivo direct plant inoculation bioassays with lower dose of** ***R. solani*** **.**
(DOCX)

Table S3 ***P*** **values of in vivo direct plant inoculation bioassays with higher dose of** ***R. solani*** **.**
(DOCX)

Table S4 ***P*** **values of in vitro plant leaf inoculation assay with** ***S.homoeocarpa.***
(DOCX)

Table S5 ***P*** **values of in vivo direct plant inoculation bioassays with** ***S. homoeocarpa.***
(DOCX)
