# Supplementary material for: Expression of a Novel Antimicrobial Peptide Penaeidin4-1 in Creeping Bentgrass (Agrostis stolonifera L.) Enhances Plant Fungal Disease Resistance
Source: PLoS One. 2011 Sep 12;6(9):e24677. doi: 10.1371/journal.pone.0024677 (PMC3171467; doi:10.1371/journal.pone.0024677)
Supplement: Table S1 — P values of in vitro plant leaf inoculation assay with R. solani . (DOCX) [file pone.0024677.s001.docx]

| **2 DPI** | |  |
| --- | --- | --- |
| **Level-Level** | | ***P* value** |
| WT | TG-1 | 0.2622 |
| WT | TG-2 | 0.9782 |
| WT | TG-3 | 0.5189 |
| WT | TG-4 | 0.7804 |
| TG-1 | TG-2 | 0.5865 |
| TG-1 | TG-3 | 0.99 |
| TG-1 | TG-4 | 0.8967 |
| TG-2 | TG-3 | 0.8521 |
| TG-2 | TG-4 | 0.9782 |
| TG-3 | TG-4 | 0.9926 |
|  |  |  |
| **8 DPI** | |  |
| **Level-Level** | | ***P* value** |
| WT | TG-1 | 0.0753 |
| WT | TG-2 | 0.7489 |
| WT | TG-3 | 0.0091 |
| WT | TG-4 | 0.9098 |
| TG-1 | TG-2 | 0.5917 |
| TG-1 | TG-3 | 0.9213 |
| TG-1 | TG-4 | 0.3827 |
| TG-2 | TG-3 | 0.1677 |
| TG-2 | TG-4 | 0.9969 |
| TG-3 | TG-4 | 0.0814 |
|  |  |  |
| **14 DPI** | |  |
| **Level-Level** | | ***P* value** |
| WT | TG-1 | <0.001 |
| WT | TG-2 | <0.001 |
| WT | TG-3 | <0.001 |
| WT | TG-4 | <0.001 |
| TG-1 | TG-2 | 0.9263 |
| TG-1 | TG-3 | 0.9998 |
| TG-1 | TG-4 | 1 |
| TG-2 | TG-3 | 0.9662 |
| TG-2 | TG-4 | 0.955 |
| TG-3 | TG-4 | 1 |
