# Supplementary material for: Expression of a Novel Antimicrobial Peptide Penaeidin4-1 in Creeping Bentgrass (Agrostis stolonifera L.) Enhances Plant Fungal Disease Resistance
Source: PLoS One. 2011 Sep 12;6(9):e24677. doi: 10.1371/journal.pone.0024677 (PMC3171467; doi:10.1371/journal.pone.0024677)
Supplement: Table S3 — P values of in vivo direct plant inoculation bioassays with higher dose of R. solani . (DOCX) [file pone.0024677.s003.docx]

| **14 DPI** | |  |
| --- | --- | --- |
| **Level-Level** | | ***P* value** |
| WT | TG-1 | 0.0284 |
| WT | TG-3 | 0.0256 |
| WT | TG-4 | 0.0284 |
| TG-1 | TG-3 | 0.7389 |
| TG-1 | TG-4 | 0.76 |
| TG-3 | TG-4 | 0.4357 |
