# Supplementary material for: Expression of a Novel Antimicrobial Peptide Penaeidin4-1 in Creeping Bentgrass (Agrostis stolonifera L.) Enhances Plant Fungal Disease Resistance
Source: PLoS One. 2011 Sep 12;6(9):e24677. doi: 10.1371/journal.pone.0024677 (PMC3171467; doi:10.1371/journal.pone.0024677)
Supplement: Table S4 — P values of in vitro plant leaf inoculation assay with S.homoeocarpa. (DOCX) [file pone.0024677.s004.docx]

| **2 DPI** | |  |
| --- | --- | --- |
| **Level-Level** | | ***P* value** |
| WT | TG-1 | 0.0074 |
| WT | TG-2 | 0.0037 |
| WT | TG-3 | <0.001 |
| WT | TG-4 | <0.001 |
| TG-1 | TG-2 | 0.9991 |
| TG-1 | TG-3 | 0.5765 |
| TG-1 | TG-4 | 0.8576 |
| TG-2 | TG-3 | 0.7287 |
| TG-2 | TG-4 | 0.9448 |
| TG-3 | TG-4 | 0.9873 |
|  |  |  |
| **4DPI** | |  |
| **Level-Level** | | ***P* value** |
| WT | TG-1 | 0.0231 |
| WT | TG-2 | 0.054 |
| WT | TG-3 | 0.0296 |
| WT | TG-4 | 0.0379 |
| TG-1 | TG-2 | 0.9968 |
| TG-1 | TG-3 | 1 |
| TG-1 | TG-4 | 0.9996 |
| TG-2 | TG-3 | 0.9991 |
| TG-2 | TG-4 | 0.9999 |
| TG-3 | TG-4 | 1 |
|  |  |  |
| **7 DPI** | |  |
| **Level-Level** | | ***P* value** |
| WT | TG-1 | <0.001 |
| WT | TG-2 | <0.001 |
| WT | TG-3 | <0.001 |
| WT | TG-4 | <0.001 |
| TG-1 | TG-2 | 0.9997 |
| TG-1 | TG-3 | 0.9957 |
| TG-1 | TG-4 | 1 |
| TG-2 | TG-3 | 0.9997 |
| TG-2 | TG-4 | 0.9997 |
| TG-3 | TG-4 | 0.9957 |
